# Supplementary material for: Tumour Control Probability in Cancer Stem Cells Hypothesis
Source: PLoS One. 2014 May 8;9(5):e96093. doi: 10.1371/journal.pone.0096093 (PMC4014481; doi:10.1371/journal.pone.0096093)
Supplement: File S1 — Supporting Information. Including the following: (1) the details of mathematical model, (2) the parameters used to obtain the results, and (5) references cited in Supporting Information. (PDF) [file pone.0096093.s001.pdf]

## File S1: Supplementary Information

Tumour Control Probability in Cancer Stem Cells Hypothesis

Andrew Dhawan, Mohammad Kohandel, Richard Hill, Sivabal Sivaloganathan

# 1 Mathematical Model

With the goal of developing a fully stochastic model for tumor growth and treatment by radiation, we first define a hierarchy of the heterogenous cell populations within a tumor that are central for further analysis. Following Refs. [1, 2], we consider three populations, stem $S$, progenitor $P$, and mature $M$ cells. Fundamentally, stem cells differentiate into progenitor cells, which differentiate into mature cells, or $S \to P \to M$. However, we note that while stem cells have the capacity for unlimited division, progenitor cells divide only a limited number of times, and mature cells do not divide. Thus, we assume that a progenitor cell divides exactly $N$ times before finally differentiating into a mature cell $M$. That is, we have the modified hierarchy $S \to P_1 \to \ldots \to P_N \to M$ [1, 2]. Additionally, we note that any type of cell may be killed due to radiation, and we assume that it occurs with rate $\Gamma_i$, for $i = s, p, m$, representing stem, progenitor, and mature cells, respectively. The model includes the following division pathways (note that $r_1 + r_2 + r_3 = 1$, and $i = 1, \ldots, N$):

$$
\begin{array}{rl}
S \to S + S : \rho_s r_1 & P_i \to P_{i+1} + P_{i+1} : \rho_{p_i} \\
S \to S + P_1 : \rho_s r_2 & P_N \to M + M : \rho_{p_N} \\
S \to P_1 + P_1 : \rho_s r_3 & P_i \to \emptyset : \Gamma_p \\
S \to \emptyset : \Gamma_s & M \to \emptyset : \Gamma_m
\end{array}
$$

Here $\rho_s$ and $\rho_{p_i}$ refer to the proliferation rates for stem and progenitor cells, respectively, and $r_1, r_2, r_3$ refer to the probabilities of each type of stem cell division (symmetric self-renewal, asymmetric self-renewal, or symmetric commitment). For the purposes of this model, as well as the subsequent analysis, we assume that the cell deaths are independent of one another.

In order to compute the TCP, we first define the joint probability function for the system. That is, we define the probability that the system contains a given number of each type of cell at the time $t$ [3]. We make the assumption that at the initial time $t_0$, the number of each type of cell is known and these values are denoted $n_S^0, n_{P_1}^0, \ldots, n_{P_N}^0, n_M^0$, for stem cells, each of the generations of progenitor cells, and mature cells, respectively. We denote this probability function by $p_{n_S, n_{P_1}, n_{P_2}, \ldots, n_{P_N}, n_M}(t)$. The corresponding master equation [3] is then given by:

$$
\begin{aligned}
\frac{dp}{dt} &= p_{n_S-1}\rho_s r_1(n_S - 1) + \rho_s r_2 n_S p_{n_{P_1}-1} \\
&+ \rho_s r_3(n_S + 1)p_{n_S+1, n_{P_1}-2} + \Gamma_s(n_S + 1)p_{n_S+1} \\
&- (\rho_s + \Gamma_s)n_S p \\
&+ \sum_{i=1}^{N-1} \rho_{P_i}(n_{P_i} + 1)p_{n_{P_i}+1, n_{P_{i+1}}-2} \\
&+ \sum_{i=1}^{N-1} (n_{P_i} + 1)p_{n_{P_i}+1}\Gamma_p - (\rho_{P_i} + \Gamma_p)n_{P_i} p \\
&+ \rho_{P_N}(n_{P_N} + 1)p_{n_{P_N}+1, n_M-2} - \rho_{P_N} n_{P_N} p \\
&+ \Gamma_m(n_M + 1)p_{n_M+1} - \Gamma_m n_M p \ . \qquad (1)
\end{aligned}
$$

We have omitted indices of $p(t)$ that remain unchanged for brevity. The initial condition is given by ($\delta_{i,j}$ is the Kronecker delta function):

$$p_{n_S,n_{P_1},n_{P_2},\ldots,n_{P_N},n_M}(t_0) = \delta_{n_S,n_S^0}\delta_{n_{P_1},n_{P_1}^0}\ldots\delta_{n_{P_N},n_{P_N}^0}\delta_{n_M,n_M^0} \ . \tag{2}$$

From this, we can obtain master equations for the probability functions for the number of stem cells only, as well as for the number of marker-positive cells ($S$, $P_1$, $P_2$, ..., $P_k$). Denoting the probability function for the number of stem cells, as $u_{n_S}(t)$, we observe that, by definition:

$$u_{n_S}(t) = \sum_{n_{P_1},\ldots,n_{P_N},n_M \geq 0} p_{n_S,n_{P_1},n_{P_2},\ldots,n_{P_N},n_M}(t)$$

Thus we obtain

$$\begin{aligned} \frac{du_{n_S}}{dt} &= u_{n_S-1}\rho_s r_1(n_S-1) \\ &- u_{n_S}n_S(\rho_s r_1 + \rho_s r_3 + \Gamma_s) \\ &+ u_{n_S+1}(\rho_s r_3 + \Gamma_s)(n_S+1) \ , \end{aligned} \tag{3}$$

with the initial condition $u_{n_S}(t_0) = \delta_{n_S,n_S^0}$. Following Van Kampen [3], and Zaider and Minerbo [5], we solve for $u_{n_S}(t)$ analytically by introducing the generating function $U(s,t) = \sum_{i=0}^{\infty} u_i(t)s^i$. We then obtain the following partial differential equation(PDE) for $U(s,t)$:

$$\frac{\partial U}{\partial t} - \frac{\partial U}{\partial s}(s-1)(\rho_s r_1 s - \rho_s r_3 - \Gamma_s) = 0 \ . \tag{4}$$

This PDE can be solved via the method of characteristics [4], along with the initial condition $U(s,0) = s^{n_S^0}$. Assuming that all parameters are constant, we obtain

$$U(s,t) = \left[\frac{(s-1)(\rho_s r_3 + \Gamma_s)e^{(\rho_s(r_1-r_3)-\Gamma_s)t} - \rho_s r_1 s + \rho_s r_3 + \Gamma_s}{(s-1)(\rho_s r_1)e^{(\rho_s(r_1-r_3)-\Gamma_s)t} - \rho_s r_1 s + \rho_s r_3 + \Gamma_s}\right]^{n_S^0} \ . \tag{5}$$

From this, we observe that the probability that there are no stem cells remaining at time $t$ is given by

$$U(0,t) = \left[\frac{(\rho_s r_3 + \Gamma_s)e^{(\rho_s(r_1-r_3)-\Gamma_s)t} - \rho_s r_3 - \Gamma_s}{(\rho_s r_1)e^{(\rho_s(r_1-r_3)-\Gamma_s)t} - \rho_s r_3 - \Gamma_s}\right]^{n_S^0} \ . \tag{6}$$

We thus define this quantity to be the theoretical TCP, denoted $\text{TCP}_S(t)$.

Using the same technique, we can obtain the probability function for the number of marker positive cells, denoted $v_{n_S,n_{P_1},\ldots,n_{P_k}}(t)$. We have:

$$v_{n_S,n_{P_1},\ldots,n_{P_k}}(t) = \sum_{n_{P_{k+1}},\ldots,n_{P_N},n_M \geq 0} p_{n_S,n_{P_1},n_{P_2},\ldots,n_{P_N},n_M}(t) \ . \tag{7}$$

This gives (where again we have omitted the indices which remain unchanged, for brevity):

$$
\begin{aligned}
\frac{dv}{dt} &= v_{n_S-1}\rho_s r_1(n_S-1) + \rho_s r_2 n_S v_{n_{P_1}-1} \\
&+ \rho_s r_3(n_S+1)v_{n_S+1,n_{P_1}-2} \\
&+ \Gamma_s(n_S+1)v_{n_S+1} - (\rho_s+\Gamma_s)n_S v \\
&+ \sum_{i=1}^{k-1}\rho_{P_i}(n_{P_i}+1)v_{n_{P_i}+1,n_{P_{i+1}}-2} \\
&+ \sum_{i=1}^{k-1}(n_{P_i}+1)v_{n_{P_i}+1}\Gamma_p - (\rho_{P_i}+\Gamma_p)n_{P_i}v \\
&+ (\Gamma_p+\rho_{P_k})(v_{n_{P_k}+1}(n_{P_k}+1) - v n_{P_k}) \ . \qquad (8)
\end{aligned}
$$

The initial condition is $v_{n_S,n_{P_1},\ldots,n_{P_k}}(t_0) = \delta_{n_S,n_S^0}\delta_{n_{P_1},n_{P_1}^0}\ldots\delta_{n_{P_k},n_{P_k}^0}$. To solve for $v(t)$, similar to the previous case, we introduce the generating function,

$$
V(x_S, x_1, \ldots, x_k, t) = \sum_{i_S,i_1,\ldots,i_k\geq 0} v_{i_S,i_1,\ldots,i_k}(t)x_S^{i_S}x_1^{i_1}\ldots x_k^{i_k} \ . \qquad (9)
$$

We can obtain the following PDE for $V(x_S, x_1, \ldots, x_k, t)$:

$$
\begin{aligned}
&\frac{\partial V}{\partial x_S}\left(x_S^2\rho_s r_1 - (\rho_s+\Gamma_s-\rho_s r_2 x_1)x_S\right) \\
+\ &\frac{\partial V}{\partial x_S}\left(\rho_s r_3 x_1^2 + \Gamma_s\right) \\
+\ &\left(\sum_{l=1}^{k-1}\frac{\partial V}{\partial x_l}\left(\rho_{P_l}x_{l+1}^2 - (\rho_{P_l}+\Gamma_p)x_l + \Gamma_p\right)\right) \\
+\ &\frac{\partial V}{\partial x_k}(\Gamma_p+\rho_{P_k})(1-x_k) = \frac{\partial V}{\partial t} \ , \qquad (10)
\end{aligned}
$$

with the initial condition $V(x_S, x_1, \ldots, x_k, 0) = x_S^{n_S^0}x_1^{n_{P_1}^0}\ldots x_k^{n_{P_k}^0}$. This PDE can be numerically solved via a modified method of characteristics, which is outlined in Dhawan et al. [6].

# 2 Results

The model defining the effects of radiation on the cell populations is essentially the same as that defined in [7]. Other model parameters were taken from [8], except for $\beta_s, \beta_p$, which were calculated from data collected in [7], and $\omega$, which was estimated specifically for glioblastoma treatment. The parameters used are as follows: $\rho_s = 0.6931$ (1/day), $\rho_p = \rho_{p_i} = 0.6931$ (1/day), $r_1 = 0.15$, $r_2 = 0.7$, $r_3 = 0.15$, $\alpha_s = 0.2\,\mathrm{Gy}^{-1}$, $\beta_s = 0.02\,\mathrm{Gy}^{-2}$, $\alpha_p = 0.2\,\mathrm{Gy}^{-1}$, $\beta_p = 0.02\,\mathrm{Gy}^{-2}$, $n_S^0 = 100, n_{P_1}^0 = 100$, $n_{P_2}^0 = 100$, $n_{P_3}^0 = 100$, $\omega = 15\,\mathrm{min}$, and $N = 7$. The hazard function, a function describing radiation-induced cell death, is based on the linear-quadratic (LQ) model for cell survival for each dose fraction [5]. Thus, the hazard function for an individual dose fraction of radiation, with dose $d$ and irradiation period $\omega$, takes value:

$$
f_j(t,d) = \frac{(\alpha_j + 2\beta_j d)\cdot d}{\omega} \ , \qquad (11)
$$

during the period of irradiation ($0 \leq t \leq \omega$), and for all $t > \omega$ or $t < 0$, is identically 0 (with $j = s, p$). Using this, we define the overall function describing the rate of cell death for the entire treatment, consisting of all dose fractions as:

$$\Gamma_j(t) = \sum_i f_j(t - t_i, d_i) \ , \quad j = s, p \ . \tag{12}$$

In the above expressions, $\alpha_s$, $\alpha_p$, $\beta_s$, $\beta_p$ are parameters describing the radiosensitivities of stem ($s$) and progenitor ($p$) cells in the LQ model, $t_i$ is the time of the $i^{\text{th}}$ fraction, $d_i$ is the dose administered on the $i^{\text{th}}$ fraction, and the sum is over all fractions administered.

# References


[1] C. Turner and M. Kohandel, Investigating the Link between epithelial-mesenchymal transition and the cancer stem cell phenotype: A mathematical approach, Journal of Theoretical Biology 265: 329-335 (2010).

[2] C. Turner and M. Kohandel, Quantitative approaches to cancer stem cells and epithelial-mesenchymal transition, Seminars in Cancer Biology 22: 374-8 (2012).

[3] N. G. Van Kampen, Stochastic Processes in Physics and Chemistry, North-Holland Personal Library: Elsevier Science (2007).

[4] L. C. Evans, Partial Differential Equations, Graduate Studies in Mathematics: American Mathematical Society (1998).

[5] M. Zaider and G. N. Minerbo, Tumour control probability: A formulation applicable to any temporal protocol of dose delivery, Phys. Med. Bio. 45: 279-293 (2000).

[6] A. Dhawan, K. Kaveh, M. Kohandel and S. Sivaloganathan, Stochastic model for tumor control probability: Effects of cell cycle and (a)symmetric proliferation, Submitted to Phys. Med. Bio. (2013).

[7] G. Powathil, M. Kohandel, S. Sivaloganathan, A. Oza and M.Milosevic, Modeling the spatial mathematical modeling of brain tumors: Effects of radiotherapy and chemotherapy, Phys Med Biol. 52: 3291-306 (2007).

[8] C. Turner, A. R. Stinchcombe, M. Kohandel, S. Singh and S. Sivaloganathan, Characterization of brain cancer stem cells: A mathematical approach, Cell Proliferation 42: 529-540 (2009).
